# Pericardial adiposity is independently linked to adverse cardiovascular phenotypes: a CMR study of 42 598 UK Biobank participants^[Author-notes jeac101-FM3]^

**DOI:** 10.1093/ehjci/jeac101

**Published:** 2022-05-31

**Authors:** Maddalena Ardissino, Celeste McCracken, Andrew Bard, Charalambos Antoniades, Stefan Neubauer, Nicholas C Harvey, Steffen E Petersen, Zahra Raisi-Estabragh

**Affiliations:** National Heart and Lung Institute, Imperial College London, Hammersmith Hospital, London W12 0NN, UK; William Harvey Research Institute, NIHR Barts Biomedical Research Centre, Queen Mary University of London, Charterhouse Square, London EC1M 6BQ, UK; Division of Cardiovascular Medicine, Radcliffe Department of Medicine, University of Oxford, National Institute for Health Research Oxford Biomedical Research Centre, Oxford University Hospitals NHS Foundation Trust, Oxford OX3 9DUUK; William Harvey Research Institute, NIHR Barts Biomedical Research Centre, Queen Mary University of London, Charterhouse Square, London EC1M 6BQ, UK; Division of Cardiovascular Medicine, Radcliffe Department of Medicine, University of Oxford, National Institute for Health Research Oxford Biomedical Research Centre, Oxford University Hospitals NHS Foundation Trust, Oxford OX3 9DUUK; Acute Vascular Imaging Centre, Radcliffe Department of Medicine, University of Oxford, Oxford OX1 2JD, UK; Division of Cardiovascular Medicine, Radcliffe Department of Medicine, University of Oxford, National Institute for Health Research Oxford Biomedical Research Centre, Oxford University Hospitals NHS Foundation Trust, Oxford OX3 9DUUK; MRC Lifecourse Epidemiology Centre, University of Southampton, Southampton SO16 6YD, UK; NIHR Southampton Biomedical Research Centre, University of Southampton and University Hospital Southampton NHS Foundation Trust, Southampton SO16 6YDUK; William Harvey Research Institute, NIHR Barts Biomedical Research Centre, Queen Mary University of London, Charterhouse Square, London EC1M 6BQ, UK; Barts Heart Centre, St Bartholomew’s Hospital, Barts Health NHS Trust, West Smithfield EC1A 7BE, UK; Health Data Research UK, London, UK; Alan Turing Institute, London, UK; William Harvey Research Institute, NIHR Barts Biomedical Research Centre, Queen Mary University of London, Charterhouse Square, London EC1M 6BQ, UK; Barts Heart Centre, St Bartholomew’s Hospital, Barts Health NHS Trust, West Smithfield EC1A 7BE, UK

**Keywords:** pericardial fat, cardiovascular magnetic resonance, left ventricle, left atrium, arterial stiffness, cardiometabolic disease

## Abstract

**Aims:**

We evaluated independent associations of cardiovascular magnetic resonance (CMR)-measured pericardial adipose tissue (PAT) with cardiovascular structure and function and considered underlying mechanism in 42 598 UK Biobank participants.

**Methods and results:**

We extracted PAT and selected CMR metrics using automated pipelines. We estimated associations of PAT with each CMR metric using linear regression adjusting for age, sex, ethnicity, deprivation, smoking, exercise, processed food intake, body mass index, diabetes, hypertension, height cholesterol, waist-to-hip ratio, impedance fat measures, and magnetic resonance imaging abdominal visceral adiposity measures. Higher PAT was independently associated with unhealthy left ventricular (LV) structure (greater wall thickness, higher LV mass, more concentric pattern of LV hypertrophy), poorer LV function (lower LV global function index, lower LV stroke volume), lower left atrial ejection fraction, and lower aortic distensibility. We used multiple mediation analysis to examine the potential mediating effect of cardiometabolic diseases and blood biomarkers (lipid profile, glycaemic control, inflammation) in the PAT-CMR relationships. Higher PAT was associated with cardiometabolic disease (hypertension, diabetes, high cholesterol), adverse serum lipids, poorer glycaemic control, and greater systemic inflammation. We identified potential mediation pathways via hypertension, adverse lipids, and inflammation markers, which overall only partially explained the PAT-CMR relationships.

**Conclusion:**

We demonstrate association of PAT with unhealthy cardiovascular structure and function, independent of baseline comorbidities, vascular risk factors, inflammatory markers, and multiple non-invasive and imaging measures of obesity. Our findings support an independent role of PAT in adversely impacting cardiovascular health and highlight CMR-measured PAT as a potential novel imaging biomarker of cardiovascular risk.

## Graphical Abstract

**Graphical Abstract:**
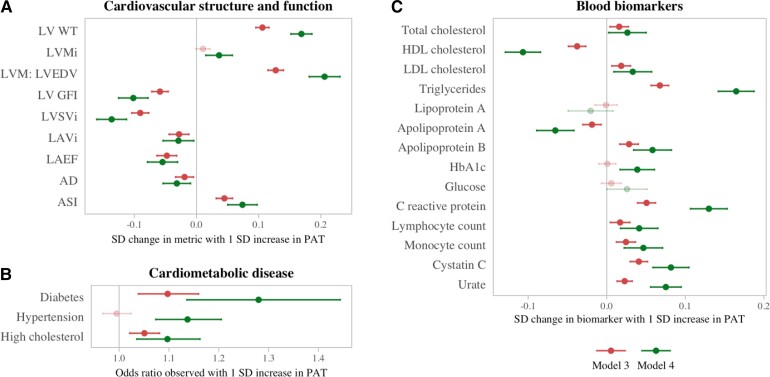
Associations of PAT with cardiovascular structure and function (A), cardiometabolic disease (B), and blood biomarkers (C). The red lines represent the model adjusted for vascular risk factors, demographic factors, body mass index and waist-hip ratio (Model 3); the green lines represent the model adjusted for vascular risk factors, demographic factors, and adiposity PCA scores (Model 4). Panel A: SD change in cardiovascular measure per SD increase in (log) PAT. Panel B: Change in cardiometabolic disease odds per SD increase in (log) PAT. Panel C: SD change in blood biomarker level per SD increase in (log) PAT. AD, aortic distensibility; ASI, arterial stiffness index; CRP, C-reactive protein; HbA1c, glycated haemoglobin A1C; HDL, high-density lipoprotein; LAEF, left atrium ejection fraction; LAVi, left atrial maximum area index; LDL, low-density lipoprotein; LV GFI, left ventricular global function index; LVMi, left ventricular mass index; LVM:LVEDV, ratio of left ventricular mass to left ventricular end-diastolic volume; LVSVi, left ventricular stroke volume index; LV WT, left ventricular wall thickness; PAT, pericardial adipose tissue, PCA, principal component analysis; SD, standard deviation

## Introduction

Pericardial adipose tissue (PAT) is the visceral adipose compartment surrounding the heart and coronary vasculature. Greater pericardial adiposity has been linked to higher risk of atrial fibrillation (AF),^1,2^ heart failure,^3^ ischaemic heart disease (IHD),^4^ and adverse left ventricular (LV) remodelling.^5^

PAT mirrors systemic inflammation,^2,6^ which is a risk factor for cardiovascular disease.^7^ Furthermore, being an adipose tissue compartment, PAT is usually associated with greater general obesity, which similarly associates with multiple vascular risk factors and cardiovascular diseases. However, the mechanistic pathways linking PAT to cardiovascular disease are incompletely understood. Importantly, it is unclear whether measurement of pericardial fat provides independent information about individuals’ cardiovascular health, over other measures of obesity, vascular risk factors, or inflammation markers.

Cardiovascular magnetic resonance (CMR) is the reference standard for evaluation of cardiac structure and function. However, until now, the absence of rapid PAT quantification methods has limited studies using this modality. We recently developed a fully automated quality-controlled tool for CMR PAT measurement,^8^ which has been used to extract PAT measurements from 42 598 CMR studies in the UK Biobank, a highly detailed biomedical research resource including clinical, imaging, and blood biochemistry data.

In this study, we first describe novel associations of CMR PAT with measures of cardiovascular structure and function. We examine the independence of these relationships from classic vascular risk factors and other measures of obesity- including anthropometric measures, impedance fat measures, and magnetic resonance imaging (MRI) measures of abdominal visceral adiposity. Second, we use multiple mediation analysis to formally evaluate the role of multiple putative mediators in any observed associations between PAT and cardiovascular phenotypes, including cardiometabolic profile and blood markers of systemic inflammation.

## Methods

### Setting and study population

The UK Biobank is a population-based cohort study including over 500 000 participants aged 40–69 years, recruited between 2006 and 2010. The protocol is publicly available.^9^ The UK Biobank Imaging Study, which was launched in 2015 and is ongoing, aims to scan a random 20% (*n* = 100 000) subset of the original participants and includes multiorgan MRI of the heart, brain, and abdomen.

### Cardiovascular structure and function

CMR examinations were performed on 1.5 Tesla scanners (MAGNETOM Aera, Syngo Platform VD13A, Siemens Healthcare, Erlangen, Germany) in dedicated imaging units according to pre-defined protocols.^10^ CMR indices were derived using a fully automated image analysis pipeline.^11^ The following measures were included in this study: LV wall thickness, LV mass, LV mass to LV end-diastolic volume ratio (LVM: LVEDV), LV global function index (LVGFI), LV stroke volume (LVSV), LA volume, LA ejection fraction (LAEF).

We considered aortic distensibility (AD) and arterial stiffness index (ASI) as measures of arterial health. AD is a CMR-derived measure of local aortic stiffness, which was extracted using an automated pipeline.^11^ ASI is a measure of large artery stiffness derived from finger plethysmography pulse waveform; recorded as per pre-defined UK Biobank protocols.

### PAT quantification

**Figure 1:**
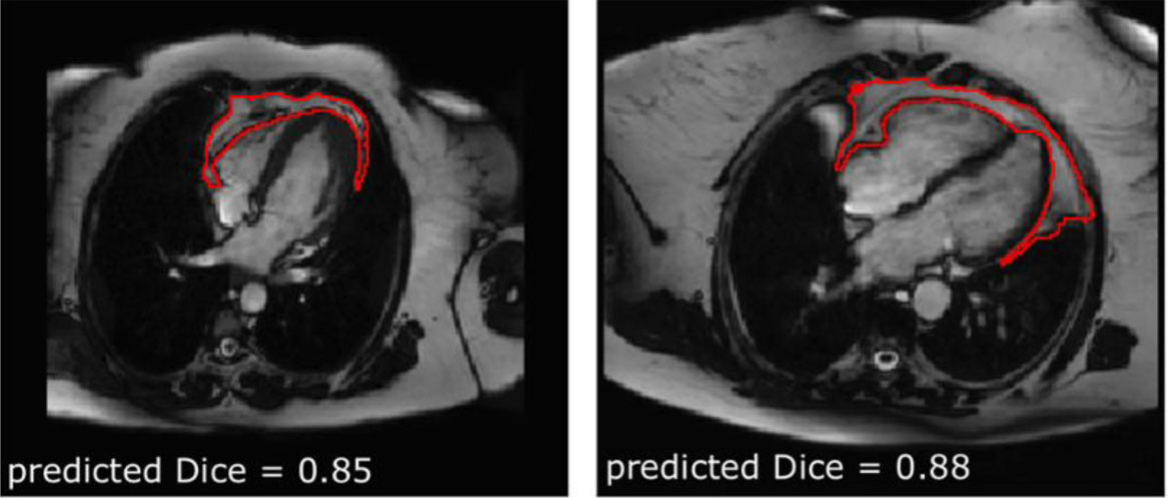
Example automated PAT segmentations and their predicted segmentation quality. The Dice score provides a quality-control measure with scores <0.7 indicating poor segmentation quality. Figure adapted from [8].

PAT area was extracted from CMR 4-chamber cine images in end-diastole using an automated tool previously developed and validated in the UK Biobank and in an external cohort.^8^ In brief, the tool comprises a neural network trained for fully automated PAT segmentation using a multi-residual U-net architecture and includes an in-built quality-control feature, which uses Dice scores as a measure of segmentation quality (*Figure 1*). The segmented area is an en-bloc 2D measure, which includes both the epicardial and pericardial fat compartments. In previous validation of this metrics, we demonstrated its correlation to more established cardiac computed tomography (CCT) measures of PAT volume and to diabetes status.^8^ The major advantage of this CMR-derived PAT area is its potential for wide application to existing routine care CMR scan, as the four-chamber slice from which it is extracted is a standard component of almost all CMR protocols and is typically acquired with minimal variability compared with other slice acquisitions. Previous attempts at measurement of PAT volume using CMR have required dedicated acquisitions (e.g. short-axis stack with no interslice gap), which preclude applicability to routine scans. Furthermore, the automation of such approaches would have limited generalisability due to high variations in slice thickness and interslice gap acquisition parameters within and between centres. Indeed, there are currently no widely available methods for automated extraction of PAT volume from standard CMR scans.

### Measures of obesity

A key aim of the study was to determine whether the relationship between PAT and cardiovascular phenotypes was distinct from other obesity measures. We considered anthropometric measures of obesity, impedance fat measures, and abdominal MRI-derived measures of visceral and subcutaneous adiposity. Body mass index (BMI) and waist-to-hip ratio (WHR) were calculated from UK Biobank body size measures. Bioelectrical impedance measures of obesity were derived using the Tanita BC418MA body composition analyser as per UK Biobank protocols.^12^ We included whole body fat mass and trunk fat mass impedance measures. From the abdominal MRI (available for 7664 participants), we selected abdominal subcutaneous, visceral adipose tissue, and total adipose tissue volume measures.^13^

### Demographics and lifestyle

We obtained sex and ethnicity from self-report at baseline assessment. Age was as recorded at imaging. The Townsend score is reported as a measure of deprivation by the UK Biobank.^14^ Smoking status was based on self-report at imaging. We took self-reported processed food intake as an indicator of diet quality. We derived a continuous value for the amount of physical activity measured in metabolic equivalent (MET) min/week.^15^

### Cardiometabolic diseases

We considered diabetes, hypertension, and high cholesterol as key cardiometabolic diseases. Diagnoses were ascertained from a combination of self-report, blood biochemistry, and linked Hospital Episode Statistics data (see Supplementary data online, *Table 1*).

### Blood biomarkers

The following blood biomarkers (measured at baseline) were included based on biological plausibility of their mechanistic role in the relationships of PAT with cardiovascular health: total cholesterol, high-density lipoprotein cholesterol (HDL), low-density lipoprotein cholesterol (LDL), triglycerides, lipoprotein A, glycated haemoglobin A1c (HbA1c), glucose, C-reactive protein (CRP), albumin, Cystatin C, urate, white cell count.

### Statistical analysis

Statistical analysis was with R statistical software version 4.1.0 (R Core Team) and RStudio version 1.4.1717 (RStudio). We estimated the association between PAT and cardiovascular phenotypes using multivariable linear regression models. PAT was set as the exposure of interest, and the cardiovascular phenotypes were (in turn) set as the model outcome (response) variable.

Model covariates were selected based on their potential confounding or mediating role after review of the literature and consideration of biological plausibility. We first modelled relationships whilst adjusting for demographic and lifestyle factors (Model 1), including age, sex, ethnicity, Townsend score, smoking, physical activity, and processed meat intake. In subsequent models, we evaluated the influence of additional adjustment for obesity measures and cardiometabolic morbidities. In Model 2, we added BMI and WHR to the set of initial covariates. In Model 3, we additionally included diabetes, hypertension, and high cholesterol along with the covariates from Models 1 and 2. Finally, in Model 4, we applied an enriched definition of body fat, by combining BMI and WHR with the impedance and abdominal MRI-derived measures of body fat. Since there were significant correlations between these body fat measures (see Supplementary data online, *Table 2A*) we performed a principal component analysis, which extracted three body fat principal components (PCs): total, visceral, and pericardial (see Supplementary data online, *Table 2B*). With this formulation, the PAT PC retains 90% of its original variance, but it is de-confounded from the other forms of body fat using the most detailed information available. The PCs were then included simultaneously into Model 4, along with all previously mentioned covariates.

We report results as standardised beta coefficients; that is, standard deviation (SD) change in outcome per SD increase in log PAT area (cm^2^) with corresponding 95% confidence intervals (CIs) and *P*-values. For Model 4, the coefficient relates to a 1 SD increase in the pericardial adiposity PC. *P*-values were corrected for multiple testing across exposures (per set of outcomes) using a false discovery rate of 0.05. PAT areas in the sample had a right-skewed distribution and were therefore log-transformed for linear modelling.

We investigated the potential mediation of the associations between PAT and cardiovascular phenotypes through cardiometabolic diseases and the selected blood biomarkers. Putative mediators were selected for this analysis based on (i) prior knowledge on their association with PAT which was considered likely to be causal in the direction from PAT to the mediator,^16,17^ and (ii) their likely causal effect on cardiovascular phenotypes.^18^ For this, we first estimated the association of PAT with each potential mediating variable using logistic regression and linear regression as appropriate, adjusting for covariates as before (Models 1–4). Variables that showed significant relationships in these analyses were taken forward for multiple mediation analysis. To simultaneously model multiple mixed mediators (continuous and binary), we performed mediation analyses using the *mmabig* package in R,^19^ with confidence intervals estimated via *n* = 400 bootstrapped samples.

## Results

### Baseline characteristics

PAT measurements were available for 45 519 participants; we excluded 2590 (5.7%) studies due to poor segmentation quality (Dice score <0.6). A further 331 participants were excluded due to missing covariates. The remaining 42 598 participants were included in the analysis (Table 1 and see Supplementary data online, *Figure 1*).

**Table 1:** Sample characteristics

	Whole set	Men	Women	Sample size
	(*n* = 42 598)	(*n* = 20 675, 49%)	(*n* = 21 923, 51%)	
Demographics and risk factors				
Age (years)	64.1 ( ± 7.7)	64.8 ( ± 7.8)	63.4 ( ± 7.5)	42 598
Caucasian (White) ethnicity	41 347 (97.1%)	20 050 (97.0%)	21 297 (97.1%)	
Body mass index (kg/m^2^)	26.0 (23.6, 28.9)	26.6 (24.4, 29.1)	25.4 (22.9, 28.6)	
Waist-to-hip ratio	0.88 ( ± 0.09)	0.94 ( ± 0.06)	0.82 ( ± 0.07)	
Townsend score	−2.6 (−3.9, −0.4)	−2.7 (−3.9, −0.5)	−2.5 (−3.8, −0.4)	
Physical activity (MET-minutes/week)	1930 (924, 3599)	1977 (967, 3637)	1893 (873, 3564)	
Smoker (current)	1480 (3.5%)	849 (4.1%)	631 (2.9%)	
Processed meat intake (g/day)	11 (5, 32)	11 (5, 32)	5 (5, 11)	
Hypertension	14 436 (33.9%)	8499 (41.1%)	5937 (27.1%)	
Diabetes	2625 (6.2%)	1678 (8.1%)	947 (4.3%)	
High cholesterol	15 321 (36.0%)	8926 (43.2%)	6395 (29.2%)	
Blood markers				
Total cholesterol (mmol/L)	5.73 ( ± 1.09)	5.59 ( ± 1.08)	5.86 ( ± 1.07)	41 750
HDL cholesterol (mmol/L)	1.47 ( ± 0.38)	1.30 ( ± 0.30)	1.63 ( ± 0.37)	
LDL cholesterol (mmol/L)	3.58 ( ± 0.83)	3.56 ( ± 0.83)	3.60 ( ± 0.83)	
Triglycerides (mmol/L)	1.41 (1.00, 2.04)	1.64 (1.16, 2.36)	1.23 (0.91, 1.73)	
Lipoprotein A (nmol/L)	20.4 (9.4, 60.8)	19.8 (9.3, 63.3)	20.9 (9.6, 58.8)	
HbA1c (mmol/mol)	34.6 (32.3, 37.1)	34.7 (32.4, 37.2)	34.5 (32.2, 37.0)	
Glucose (mmol/L)	4.88 (4.56, 5.23)	4.91 (4.57, 5.27)	4.86 (4.55, 5.19)	
C-reactive protein (mg/L)	1.09 (0.56, 2.19)	1.09 (0.57, 2.05)	1.10 (0.55, 2.33)	
Cystatin C (mg/L)	0.86 (0.79, 0.95)	0.90 (0.83, 0.98)	0.83 (0.76, 0.91)	
Urate (umol/L)	304.9 ( ± 77.7)	350.8 ( ± 67.0)	261.5 ( ± 60.1)	
Apolipoprotein A (g/L)	1.55 ( ± 0.26)	1.44 ( ± 0.22)	1.65 ( ± 0.26)	
Apolipoprotein B (g/L)	1.03 ( ± 0.23)	1.04 ( ± 0.23)	1.02 ( ± 0.23)	
Lymphocyte count (10^9^ cells/L)	1.82 (1.50, 2.20)	1.79 (1.46, 2.16)	1.90 (1.55, 2.30)	
Monocyte count (10^9 ^cells/L)	0.44 (0.36, 0.54)	0.49 (0.40, 0.60)	0.40 (0.32, 0.50)	
Impedance fat measures				
Whole body fat mass (kg)	22.1 (17.6, 27.7)	20.3 (16.2, 25.1)	24.0 (19.1, 30.2)	42 107
Trunk fat mass (kg)	13.1 ( ± 4.7)	13.2 ( ± 4.5)	13.0 ( ± 4.9)	
Abdominal MRI adipose metrics				
Abdominal subcutaneous adipose tissue (L)	6.46 (4.80, 8.69)	5.48 (4.28, 7.02)	7.65 (5.76, 10.07)	9 100
Total adipose tissue (L)	21.1 ( ± 7.0)	19.7 ( ± 6.4)	22.2 ( ± 7.4)	
Visceral adipose tissue (L)	3.36 (2.00, 5.09)	4.70 (3.25, 6.35)	2.38 (1.51, 3.56)	
Pericardial adipose tissue				
Pericardial fat (cm^2^)	21.4 (15.0, 30.6)	27.5 (19.5, 37.5)	17.4 (13.0, 23.6)	42 598
Left ventricle (LV)				
Wall thickness (mm)	5.70 ( ± 0.77)	6.22 ( ± 0.65)	5.22 ( ± 0.53)	30 185
LV mass indexed (g/m^2^)	46.0 ( ± 8.7)	51.4 ( ± 7.8)	40.8 ( ± 5.8)	
LVM: LVEDV	0.58 (0.52, 0.64)	0.61 (0.56, 0.67)	0.55 (0.50, 0.60)	
LVGFI (%)	47.5 ( ± 6.8)	44.7 ( ± 6.3)	50.2 ( ± 6.3)	
LVSVi (ml/m^2^)	46.8 ( ± 8.4)	48.4 ( ± 9.0)	45.2 ( ± 7.3)	
Left atrium (LA)				
LA volume indexed (ml/m^2^)	38.0 (31.5, 45.4)	38.0 (30.9, 46.0)	38.1 (31.9, 45.0)	25 283
LA ejection fraction (%)	61.2 ( ± 9.1)	60.6 ( ± 9.6)	61.9 ( ± 8.5)	
Arterial compliance				
Arterial stiffness index (m/s)	9.51 ( ± 2.73)	10.00 ( ± 2.69)	9.02 ( ± 2.68)	35 205
Aortic distensibility (10^−3 ^mmHg-1)	2.18 (1.55, 3.02)	2.20 (1.59, 2.99)	2.17 (1.52, 3.05)	21 581

HbA1c, glycated haemoglobin A1C; HDL, high-density lipoprotein cholesterol; LDL, low-density lipoprotein; LVEDV, left ventricular end-diastolic volume; LVGFI, left ventricular global function index; LVM, left ventricular mass; LVSVi, left ventricular stroke volume index; MET, metabolic equivalent task; MRI, magnetic resonance imaging.

The average age was 64.1 ( ± 7.7) years old. The sample include 21 923 (51%) women (*Table 1*). The median BMI was 26.0 (23.6, 28.9) kg/m^2^. The most prevalent cardiometabolic diseases were high cholesterol (36.0%) and hypertension (33.9%). A total of 6.2% of participants had diabetes. Cardiometabolic diseases were more prevalent in men compared to women. The median PAT area was 21.4 (15.0, 30.6) cm^2^; this was higher among men, 27.5 (19.5, 37.5) cm^2^ than women, 17.4 (13.0, 23.6) cm^2^.

### PAT and cardiovascular phenotypes

Larger PAT area was associated with adverse cardiovascular structure and function across all metrics considered. These relationships were independent of confounders, cardiometabolic diseases, and all the obesity measures considered (*Table 2*).

**Table 2:** Associations between PAT and cardiovascular structure and function metrics

	Model 1	Model 2	Model 3	Model 4
LV wall thickness	0.27^a,b^	0.11^a^	0.11^a^	0.17^a^
(mm)	(0.26, 0.28)	(0.10, 0.12)	(0.10, 0.12)	(0.15, 0.19)
	< 1.00 × 10^−300^	4.42 × 10^−83^	1.25 × 10^−84^	4.69 × 10^−84^
LV mass index (g/m^2^)	0.05^a^	0.01	0.01	0.04^a^
	(0.04, 0.06)	(−0.00, 0.02)	(−0.00, 0.02)	(0.01, 0.06)
	1.31 × 10^−22^	0.0688	0.0657	8.69 × 10^−4^
LVM: LVEDV (g/ml)	0.25^a,b^	0.13^a^	0.13^a^	0.21^a^
	(0.24, 0.26)	(0.12, 0.14)	(0.11, 0.14)	(0.18, 0.23)
	< 1.00 × 10^−300^	4.20 × 10^−90^	1.31 × 10^−88^	3.90 × 10^−59^
LVGFI (%)	−0.11^a^	−0.06^a^	−0.06^a^	−0.10^a^
	(−0.12, −0.10)	(−0.07, −0.05)	(−0.07, −0.05)	(−0.13, −0.08)
	1.35 × 10^−79^	5.19 × 10^−19^	1.91 × 10^−18^	5.61 × 10^−17^
LVSVi (ml/m^2^)	−0.15^a^	−0.09^a^	−0.09^a^	−0.14^a^
	(−0.16, −0.14)	(−0.11, −0.08)	(−0.10, −0.08)	(−0.16, −0.11)
	3.05 × 10^−135^	6.46 × 10^−40^	1.07 × 10^−38^	7.25 × 10^−29^
LA volume (ml/m^2^)	−0.00	−0.03^a^	−0.03^a^	−0.03^a^
	(−0.02, 0.01)	(−0.04, −0.01)	(−0.04, −0.01)	(−0.05, −0.00)
	0.8648	3.94 × 10^−−4^	5.08 × 10^−−4^	0.0209
LAEF (%)	−0.07^a^	−0.05^a^	−0.05^a^	−0.05^a^
	(−0.08, −0.06)	(−0.06, −0.03)	(−0.06, −0.03)	(−0.08, −0.03)
	8.52 × 10^−24^	2.04 × 10^−9^	3.63 × 10^−9^	9.76 × 10^−6^
Aortic distensibility	−0.05^a^	−0.02^a^	−0.02^a^	−0.03^a^
(10^−3^ mmHg^−1^)	(−0.07, −0.04)	(−0.03, −0.01)	(−0.03, −0.00)	(−0.05, −0.01)
	7.69 × 10^−17^	0.0081	0.0086	0.0049
Arterial stiffness index	0.09^a^	0.04^a^	0.04^a^	0.07^a^
(m/s)	(0.08, 0.10)	(0.03, 0.06)	(0.03, 0.06)	(0.05, 0.10)
	1.36 × 10^−49^	2.37 × 10^−11^	2.23 × 10^−11^	9.13 × 10^−10^

Results are the association of the PAT variable (model exposure) with each cardiovascular metric (set as model outcome) from linear regression models expressed as standardised beta coefficients (SD increase in outcome measure) per SD increase in log PAT area (cm^2^), corresponding 95% confidence intervals (CIs), and *P*-values.

a indicates a *P*-value significant with a false discovery rate of 0.05 across exposures.

b indicates a *P*-value below the reporting threshold. Model 1 covariates: age, sex, ethnicity, Townsend score, smoking, physical activity, and processed food intake. Model 2 covariates: Model 1 + body mass index and waist-to-hip ratio. Model 3 covariates: Model 2 + diabetes, hypertension, and high cholesterol. Model 4 covariates: Model 1 + diabetes, hypertension, high cholesterol and the three obesity PCs (total, visceral, pericardial) derived from all available obesity measures (see Supplementary data online, *Table 2B*). Sample sizes for Models 1–3 are between 21 581 and 35 205. For Model 4, sample sizes are between 6950 and 7562. LAEF, left atrium ejection fraction; LV GFI, left ventricular global function index; LVM LVEDV, left ventricular mass to left ventricular end-diastolic volume ratio; LVSVi, left ventricular stroke volume index; PAT, pericardial adipose tissue.

In the fully adjusted models (*Table 2*, Central illustration), larger PAT area was associated with an unhealthy pattern of LV remodelling, comprising greater wall thickness (Beta: 0.17; 95% CI: 0.15, 0.19; *P* = 4.69 × 10^−84^), higher LV mass (Beta: 0.04; 95% CI: 0.01, 0.06; *P* = 8.69 × 10^−4^), and a more concentric pattern of LV remodelling (higher LVM:LVEDV; Beta: 0.21, 95% CI: 0.18, 0.23; *P* = 3.90 × 10^−59^). Higher PAT was also linked to significantly poorer LV function, specifically lower LVGFI (Beta: −0.10, 95% CI: −0.13, −0.08; *P* = 5.61 × 10^−17^) and lower LVSVi (Beta: −0.14, 95% CI: −0.16, −0.11; *P* = 7.25 × 10^−29^).

Higher PAT was also linked to significantly poorer LA function (lower LAEF, Beta: −0.05; 95% CI: −0.08, −0.03; *P* = 9.76 × 10^−6^) and smaller LA volumes (Beta: −0.03, 95%CI: −0.05, −0.00; *P* = 0.02).

Furthermore, higher PAT was linked to poorer arterial compliance by both AD and ASI. In the fully adjusted models, larger PAT area was associated with lower AD (Beta: −0.03; 95% CI: −0.05, −0.01; *P* = 4.90 × 10^−3^) and higher ASI (Beta: 0.07; 95% CI: 0.05, 0.10; *P* = 9.13 × 10^−10^).

### PAT and cardiometabolic disease

In fully adjusted models (*Table 3*, Central illustration), greater PAT was linked to significantly higher odds of diabetes (OR: 1.28; 95% CI: 1.14, 1.44; *P* = 5.31 × 10^−5^), hypertension (OR: 1.14; 95% CI: 1.07, 1.21; *P* = 1.30 × 10^−5^), and hypercholesterolaemia (OR: 1.10; 95% CI: 1.04, 1.16; *P* = 0.0017).

**Table 3:** Associations between PAT and cardiometabolic disease

	Model 1	Model 2	Model 3	Model 4
Diabetes	1.64^a^	1.12^a^	1.10^a^	1.28^a^
	(1.57, 1.72)	(1.06, 1.18)	(1.04, 1.16)	(1.14, 1.44)
	9.05 × 10^−93^	6.41 × 10^−5^	9.35 × 10^−4^	5.31 × 10^−5^
Hypertension	1.35^a^	1.01	1.00	1.14^a^
	(1.32, 1.38)	(0.98, 1.04)	(0.97, 1.02)	(1.07, 1.21)
	1.28 × 10^−133^	0.3882	0.7344	1.30 × 10^−5^
High cholesterol	1.28^a^	1.06^a^	1.05^a^	1.10^a^
	(1.25, 1.31)	(1.03, 1.09)	(1.02, 1.08)	(1.04, 1.16)
	8.42 × 10^−90^	7.27 × 10^−5^	6.58 × 10^−4^	0.0017

Results are the association of the PAT variable (model exposure) with each disease (set as model outcome) from logistic regression models expressed as odds ratio per SD increase in log PAT area (cm^2^), corresponding 95% confidence intervals (CIs), and *P*-values.

a indicates a *P*-value significant with a false discovery rate of 0.05 across exposures. Model 1 covariates: age, sex, ethnicity, Townsend score, smoking, physical activity, and processed food intake. Model 2 covariates: Model 1 + body mass index and waist-to-hip ratio. Model 3 covariates: Model 2 + diabetes, hypertension, and high cholesterol (except for the condition which is the outcome). Model 4 covariates: Model 1 + diabetes, hypertension, high cholesterol (except for the condition which is the outcome) and the three obesity PCs (total, visceral, pericardial) derived from all available obesity measures (see Supplementary data online, *Table 2B*). Sample size for Models 1–3 is 42 598. For Model 4, sample size is 7664. PAT, pericardial adipose tissue.

### PAT and blood biomarkers

Higher PAT was consistently associated with an adverse serum lipid profile, poorer glycaemic control, and markers of systemic inflammation (see Supplementary data online, *Table 3*, Central illustration).

Larger PAT area was associated with higher total cholesterol (Beta: 0.03; 95% CI: 0.00, 0.05), lower HDL cholesterol (Beta: −0.11: 95% CI: −0.13, −0.08), higher LDL cholesterol (Beta: 0.03; 95% CI: 0.01, 0.06), higher triglycerides (Beta: 0.16; 95% CI: 0.14, 0.19), lower apolipoprotein A (Beta: −0.07; 95% CI: −0.09, −0.04), higher apolipoprotein B (Beta: 0.06; 95% CI: 0.03, 0.08), and higher HbA1c (Beta: 0.04; 95% CI: 0.02, 0.06).

PAT was positively associated with markers of systemic inflammation; specifically, CRP (Beta: 0.13; 95% CI: 0.11, 0.15), lymphocyte count (Beta: 0.04; 95% CI: 0.02, 0.07), and monocyte count (Beta: 0.05; 95% CI: 0.02, 0.07). In addition, larger PAT was linked to higher Cystatin C (Beta: 0.08; 95% CI: 0.06, 0.10), and higher urate levels (Beta: 0.08; 95% CI: 0.06, 0.09).

### Mediation analysis

**Figure 2:**
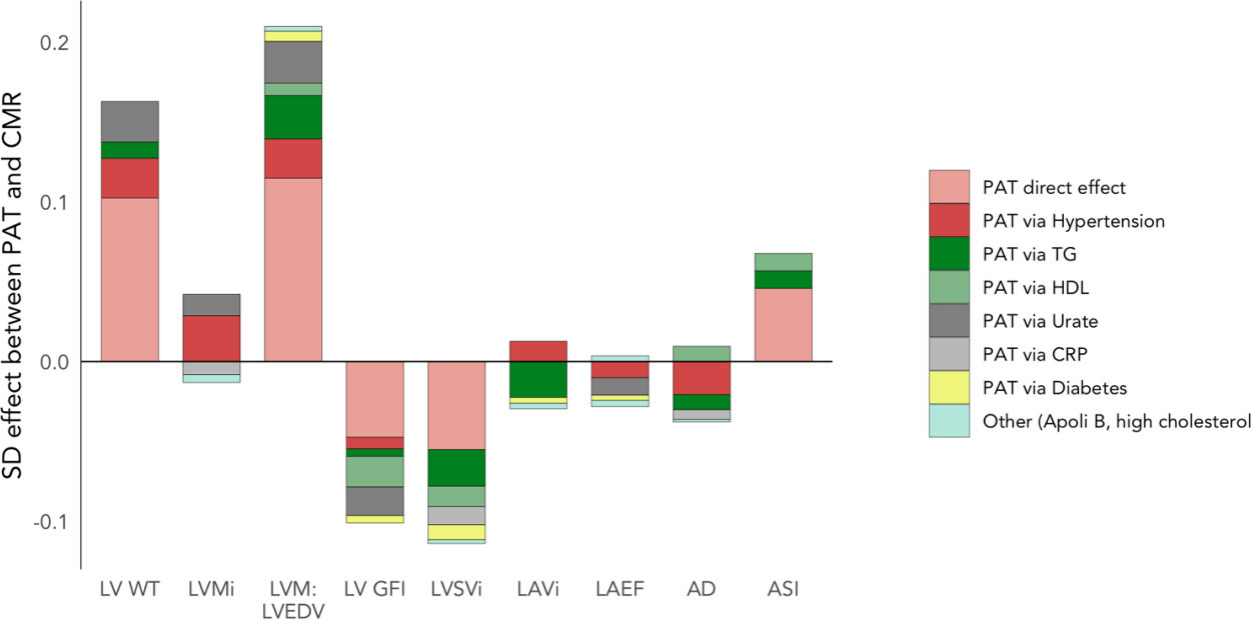
Potential mediating effects between PAT and cardiovascular phenotypes from multiple mediation analysis. Average effects from mediation analysis between PAT (the exposure) and cardiovascular metric for each of the eight mediator variables modelled together. The overall height of bars reflects the total effect between PAT and cardiac variable. Multiple mediation models were adjusted by age, sex, ethnicity, Townsend deprivation score, physical activity, processed meat intake, smoking, body mass index, and waist-hip ratio. Only effects significantly different from zero are shown, ascertained where bootstrapped confidence intervals did not contain zero, and parametric *P*-value <0.03. PAT, pericardial adipose tissue; ApoliB, Apolipoprotein B; AD, aortic distensibility; ASI, arterial stiffness index; CRP, C-reactive protein; HDL, high-density lipoprotein cholesterol; LAEF, left atrial ejection fraction; LAVi, left atrial volume index; LV, left ventricular; LV GFI, LV global function index; LVMi, LV mass index; LVSVi, LV stroke volume index; LV WT, LV wall thickness; TG, serum triglycerides.

In analyses considering mediating effect of individual variables in the relationships between PAT and cardiovascular phenotypes, across all outcomes, significant mediation effect was observed with diabetes, high cholesterol, HDL cholesterol, triglyceride level, CRP, urate, and Apolipoprotein B. We summarize PAT-CMR associations and contributions to effects from each potential mediator in *Figure 2* and see Supplementary data, *Figure 2*. Full results of the analysis are presented in Supplementary data online, *Tables 4* and *5*.

Since the main effects of PAT accumulation were increased LV wall thickness/concentricity (higher LV WT, higher LVM:LVEDV), poorer LV function (lower LVGFI, lower LVSVi), and increased arterial stiffness, we sought to investigate these effects further with mediation analysis. Across these CMR features, 47–62% of the effect of PAT on the cardiovascular system was unmediated (shown in light red in *Figure 2*), in other words, there was a significant direct effect of PAT on the heart independent of all mediators and confounders. In terms of LV wall thickness and concentricity (higher LV WT, higher LVM: LVEDV), hypertension played a significant role in mediating the effect of PAT (12–15% proportion mediated) with a similar contribution from elevated urate (12–15%), and increased triglycerides (10–13%). For LV function (LVGFI, LVSVi), the effect of PAT accumulation is primarily mediated via adverse lipid changes (higher triglycerides, and lower HDL cholesterol, 25–30% combined), with a secondary mediation through inflammatory markers (18% via urate for LVGFI, 10% via CRP for LVSVi). Partial mediation between higher PAT and higher arterial stiffness was observed via adverse lipid alterations (15% via lower HDL, and 12% via increased triglycerides). Effects of PAT on the other cardiovascular features were fully mediated by various pathways, with hypertension, triglycerides, and inflammatory markers as key potential mediators.

## Discussion

### Summary of results

In this large population-based study of 42 598 participants, we demonstrate novel associations of CMR-measured PAT with adverse cardiovascular structure and function, independent of a wide range of confounders, anthropometric measures of obesity, and MRI measures of abdominal visceral adiposity.

Specifically, larger PAT was linked with unhealthy LV structure (greater wall thickness, higher LV mass, more concentric pattern of LV remodelling), poorer LV function (lower LVGFI, lower LVSVi), poorer LA function (lower LAEF), and lower arterial compliance (lower AD, higher ASI). Additionally, we observed significant associations of higher PAT with cardiometabolic disease (hypertension, diabetes, high cholesterol) and a pattern of blood biomarker associations indicative of adverse serum lipids, poorer glycaemic control, and a proinflammatory profile. Multiple mediation analysis revealed several potential mechanistic pathways, with hypertension, lipid changes, and inflammatory biomarkers as the most important potential partial mediators. The observed CMR phenotype broadly reflected a picture of diastolic dysfunction, which is a known prominent feature of obesity-related cardiac remodelling.^20^

### Comparison with existing research

The role of PAT in long-term development of cardiovascular diseases has been a topic of growing research interest. It is well known that measures and distribution of adiposity vary greatly between individuals with some depositing more visceral fat than others, even among individuals with a normal BMI.^21^ The deposition and distribution of PAT has been associated with the risk of a host of cardiovascular diseases, such as AF,^1,2^ heart failure,^3^ and IHD.^4^ These observations are corroborated in the poorer cardiovascular parameters that we demonstrated in this study that adds to existing literature by demonstrating these association independent of other obesity measures.

A key finding that this study adds to current literature is the demonstration that an association between PAT and adverse cardiometabolic biochemical, clinical and imaging phenotype persists despite adjustment for MRI-based measures of abdominal visceral adiposity. This observation has key pathophysiological implications as it distinguishes PAT from other visceral adipose tissue stores and highlights the prognostic importance of specifically measuring PAT over other measures of obesity. Furthermore, the PAT measures in this study were acquired from routinely acquired CMR images, making the results clinically translatable.

Existing studies of CMR-measured PAT are mostly limited to small cohorts.^22,23^ Furthermore, studies of the relationships between PAT and cardiovascular phenotypes are generally sparse. In a study on a cohort of 997 Framingham Heart Study (FHS) participants who underwent chest and abdominal CT and CMR, a correlation between PAT and adverse CMR phenotypes was reported, in line with the results of our study. However, in the FHS study, multivariable analyses accounting for CT-based measures of non-pericardial adiposity suggested that the majority of the associations described were not independently related to PAT, with the notable exception of LA size.^24^ It is important to note that the FHS is smaller than the UK Biobank cohort, and that our study considers a wider variety and more robust measures of adiposity (e.g. including impedance measures and MRI-measured VAT). In a study of 145 participants with underlying cardiovascular disease and type 2 diabetes, Al-Talabany *et al*.^25^ demonstrate consistent findings to our study, reporting positive association of CMR-measured epicardial adiposity and arterial stiffness measured by pulse wave velocity. In an echocardiography study by Kim *et al.*^26^, higher PAT was associated with greater LV mass and poorer LV function by tissue doppler imaging (TDI) velocities. Similar to our study, these associations remained robust after adjustment for classic vascular risk factors, BMI, and waist circumference. Furthermore, in keeping with our observations, in a study of 4234 Multi-Ethnic Study of Atherosclerosis (MESA) participants, Shah *et al*.^5^ demonstrate association of greater PAT measured by CCT with higher LV mass and a more concentric pattern of LV hypertrophy (higher LVM: LVEDV). Similarly, in the Heinz Nixdorf Recall study, assessment of epicardial fat volume improved the prediction of incident cardiovascular events in addition to the role of coronary artery calcium scoring and established risk factor-based prediction.^27^ Finally, recent evidence has emerged linking greater epicardial^28^ and pericardial^3^ fat volumes to increased risk of incident heart failure with preserved ejection fraction (HFpEF). This carries important clinical implications, as it suggests that PAT volume assessment may widen the scope to improve HFpEF prediction.

The association between PAT and systemic inflammation has been well described in the past. In participants of the Framingham Offspring Study, CRP, tumour necrosis factor 1-alpha and urinary isoprostanates, all measures of systemic inflammation, were positively correlated with PAT even when adjusted for BMI and WHR. This mirrors the results of our study well. We identified further associations between PAT and serum cholesterol (lower HDL, higher LDL, higher total cholesterol), higher triglycerides, lower Apolipoprotein A and higher Apolipoprotein B, higher HbA1c, higher inflammatory markers (CRP, lymphocyte count, monocyte count, urate), and higher Cystatin C.

We observed a notable difference in baseline PAT between men and women. This is an interesting finding, as it may be a direct reflection of differential risk factor profiles across the sexes, differences in body composition, and/or hormonal influences. It also suggests a possible mechanistic role of PAT in the differential rates and patterns of CVD between men and women, which is an important question for further research. In terms of the implications for our results, the models in this study were adjusted for sex, and therefore we do not expect the difference across sexes to confound the results. However, it highlights the possible requirement for an indexed measure of PAT, or for sex-stratified thresholds of ‘normal ranges’ for PAT areas. This is an important step that will be required for future clinical implementation of PAT measurement.

### Potential biological mechanisms

In our mediation analysis, we demonstrated that only a small part of the association between PAT and adverse CMR phenotypes was mediated by underlying vascular risk factors, inflammatory markers, or lipid profiles. This indicates that, beyond it being a general measure of obesity, inflammation and vascular risk, PAT acts on cardiac structure and function through further independent biological mechanisms, rather than simply being a product of a common underlying disease process.

There are a number of possible explanations for this phenomenon. First, it has been suggested that the deposition of pericardial fat is higher among participants with underlying inflammatory conditions. Thus, it is possible that intrinsic inflammatory load encourages the specific deposition of PAT over other visceral, or subcutaneous fat. However, in this case we would have expected inflammatory markers to mediate the vast majority of the relationship between PAT and cardiovascular metrics in this study. This was not the case, although the panel of inflammatory markers investigated was not extensive. An important alternative explanation for this incremental effect may lie in the potential paracrine action of PAT. The highly metabolic activity of PAT is well reported; preclinical studies have demonstrated that secretion of inflammatory mediators by PAT, which can act in a paracrine manner causing localised inflammation in immediately adjacent tissues.^29^ Supporting this hypothesis, PAT is known to be distributed asymmetrically in ‘pockets’ over the myocardium^30^ and studies have demonstrated that the distribution of coronary artery disease closely follows the distribution of PAT.^6,21,31^ This supports the hypothesis that this highly metabolically active tissue may exert a degree of proinflammatory paracrine effects that accelerate atherosclerosis and regional inflammation with consequent localised damage.^1,30^

In this study, an important potential mediator of the relationship between PAT and cardiovascular structure and function was triglyceride levels. There are several possible explanations for this. Myocardial triglyceride content measured on CMR has previously been associated with higher rates of major cardiovascular events and heart failure hospitalization,^32^ but no study thus far has addressed the association between triglyceride levels and cardiac structure and function. The effect observed in this study may be directly related to intramyocardial triglyceride deposition, or may reflect the well-known correlation between obesity, metabolic syndrome, and circulating triglyceride levels. As expected, we also observed significant mediating effect of hypertension, which was particularly notable for its role in promotion of LV hypertrophy in a concentric pattern and in promoting greater aortic stiffness.

### Clinical implications

We demonstrate the value of PAT as an indicator of cardiovascular health, independent of other measures of obesity and cardiometabolic disease. Our findings support a distinct mechanistic role for PAT in driving cardiovascular disease. Furthermore, we demonstrate the potential of CMR-measured PAT as a novel imaging biomarker. The automated tool used in the present study is designed to extract PAT measurements in approximately 3 s from standard-of-care CMR scans, without need for dedicated acquisitions. As such, after appropriate validation steps, this metric could be incorporated into routine clinical workflows. It is important to note that some of the effect estimates for the associations described in this study between PAT and CMR phenotypes were statistically significant but small, and although these allow inferences about mechanistic pathways and cardiovascular risk at a population level their clinical significance for individual-level risk estimation is yet to be determined. Further work is required to demonstrate the clinical utility of CMR PAT in other independent cohorts and in association with incident health outcomes.

### Strengths and limitations

This is the largest CMR study to investigate the association between PAT and cardiovascular phenotypes and the first population-based study to explore this question. Associations between PAT and incident health events may be evaluated in coming years as outcomes accrue in the imaging cohort of the UK Biobank. A limitation of this study is that PAT was measured based on fat area only, rather than based on volumetric quantification, and the measurement included paracardial, epicardial, and pericardial fat compartments, although existing literature has identified that epicardial fat has a stronger correlation with cardiovascular risk and morbidity. However, this ties into a strength of the study, as we used an automated tool for quantification of PAT based on routinely acquired CMR images. This is an important strength for two reasons. First, it allowed the processing of a large number of images and broadens the clinical applicability of the measure. Second, and most importantly, the fact that the PAT quantification tool was built on routinely acquired CMR images makes it more clinically translatable, as it requires no dedicated imaging sequences.

A further limitation of this study is that the UK Biobank cohort is known to be healthier than the general UK population, and this may be partly reflected in this study by the right-skewed distribution of PAT area measurements. Further validation in cohorts with higher morbidity rates is therefore warranted. For this reason, we could not explore associations with LVEF, as the vast majority of patients in the UK Biobank Imaging Study have LVEFs in the normal range which limits its usefulness as a reliable indicator of health, as discussed in previous work.^33,34^

A limitation of the mediation analyses presented in this study is that the direction of the underlying causal associations between the exposure (PAT), the putative mediators and the outcome (CMR phenotypes) is not clear. Though the direction of the causal pathway from the mediators to the outcome is well established (e.g. hypertension to CMR phenotype), the direction of the potentially causal pathway between the exposure (PAT) and mediator is less clear.^35^ Depending on the direction of this causal pathway, the factors considered in this study may either be true mediators, confounders, or both if a bidirectional relationship exists. Without temporal trends and in the setting of a retrospective study, this issue could not be explored further in this cohort. For this reason, no claim on definitive mediation can be made on the basis of our results, but the potential mediators identified should be considered important targets for further investigation. Furthermore, this does not detract from the main message derived from this analysis: that there is a persistent association between PAT and CMR phenotypes that is not accounted for by incorporation of these factors in the models.

Finally, many of the CMR parameters described are interrelated (e.g. LA size and LV hypertrophy). We did not attempt to isolate or identify a ‘cardinal’ driver of unhealthy CMR phenotype with increasing PAT, as we opted to provide a holistic picture of the pattern of changes associated with PAT. Overall, our study identifies an independent mechanistic action of PAT on adverse cardiovascular phenotypes: though some of the association was explained by inflammatory markers, vascular risk factors and general obesity, a sizeable proportion was not. Dedicated studies for identification of the pathways that underlie this direct association are warranted, and future work examining the association of PAT with myocardial tissue characterization metrics—including mapping and spectroscopy, may provide additional important biologic insight into mechanisms.

## Conclusions

Our findings support an independent role of PAT in adversely impacting cardiovascular health. The results of this study encourage two avenues of research in this field: first, validation of the tools used in this study and its findings, with a view to integrate PAT measurement in routine CMR analysis as an imaging biomarker of cardiovascular risk. Second, mechanistic research to identify underlying mechanistic pathways which may mediate the observed relationships and to explore the potential scope or benefit of intervention.

## Supplementary Material

jeac101_Supplementary_Data

## Data Availability

This research was conducted using the UK Biobank resource under access application 2964. UK Biobank will make the data available to all bona fide researchers for all types of health-related research that is in the public interest, without preferential or exclusive access for any persons. All researchers will be subject to the same application process and approval criteria as specified by UK Biobank. For more details on the access procedure, see the UK Biobank website: http://www.ukbiobank.ac.uk/register-apply.

## References

[1] Wong CX , Ganesan AN, Selvanayagam JB. Epicardial fat and atrial fibrillation: current evidence, potential mechanisms, clinical implications, and future directions. Eur Heart J 2017;38:1294–302. 10.1093/eurheartj/ehw045 26935271

[2] Batal O , Schoenhagen P, Shao M, Ayyad AE, Van Wagoner DR, Halliburton SS, et al Left atrial epicardial adiposity and atrial fibrillation. Circ Arrhythmia Electrophysiol 2010;3:230–6. 10.1161/CIRCEP.110.957241 PMC2974566 20504944

[3] Kenchaiah S , Ding J, Carr JJ, Allison MA, Budoff MJ, Tracy RP, et al Pericardial fat and the risk of heart failure. J Am Coll Cardiol 2021;77:2638–52. 34045020 10.1016/j.jacc.2021.04.003 PMC8218602

[4] Greif M , Becker A, Von Ziegler F, Lebherz C, Lehrke M, Broedl UC, et al Pericardial adipose tissue determined by dual source CT is a risk factor for coronary atherosclerosis. Arterioscler Thromb Vasc Biol 2009;29:781–6. 19229071 10.1161/ATVBAHA.108.180653

[5] Shah R V , Anderson A, Ding J, Budoff M, Rider O, Petersen SE, et al Pericardial, but not hepatic, fat by CT is associated with CV outcomes and structure. JACC Cardiovasc Imaging 2017; 10:1016–27. 28330662 10.1016/j.jcmg.2016.10.024 PMC5591038

[6] Zimmermann GS , Ruether T, Von Ziegler F, Greif M, Tittus J, Schenzle J, et al Increased pericardial adipose tissue in smokers. J Clin Med 2021;10:3382. 34362164 10.3390/jcm10153382 PMC8348719

[7] Ruparelia N , Chai JT, Fisher EA, Choudhury RP. Inflammatory processes in cardiovascular disease: a route to targeted therapies. Nat Rev Cardiol 2017;14:133–44. 27905474 10.1038/nrcardio.2016.185 PMC5525550

[8] Bard A , Raisi-Estabragh Z, Ardissino M, Lee AM, Pugliese F, Dey D, et al Automated quality-controlled cardiovascular magnetic resonance pericardial fat quantification using a convolutional neural network in the UK biobank. Front Cardiovasc Med 2021;8:1–11. 10.3389/fcvm.2021.677574 PMC8294033 34307493

[9] UK Biobank Coordinating Centre . UK Biobank: Protocol for a large-scale prospective epidemiological resource. 2007. p. 1–112. https://www.ukbiobank.ac.uk/media/gnkeyh2q/study-rationale.pdf (13 December 2021, date last accessed)

[10] Petersen SE , Matthews PM, Francis JM, Robson MD, Zemrak F, Boubertakh R, et al UK Biobank’s cardiovascular magnetic resonance protocol. J Cardiovasc Magn Reson 2015;18:8. 10.1186/s12968-016-0227-4 PMC4736703 26830817

[11] Bai W , Suzuki H, Huang J, Francis C, Wang S, Tarroni G, et al A population-based phenome-wide association study of cardiac and aortic structure and function. Nat Med 2020;26:1654–62. 32839619 10.1038/s41591-020-1009-y PMC7613250

[12] UK Biobank . Body Composition Measurement. https://biobank.ndph.ox.ac.uk/showcase/ukb/docs/body_composition.pdf (13 December 2021, date last accessed)

[13] West J , Dahlqvist Leinhard O, Romu T, Collins R, Garratt S, Bell JD, et al Feasibility of MR-based body composition analysis in large scale population studies. PLoS One 2016;11:e0163332. 27662190 10.1371/journal.pone.0163332 PMC5035023

[14] Townsend P , Phillimore P, Beattie A. Health and deprivation: inequality and the North. Nurs Stand 1988;2:34–4. 10.7748/ns.2.17.34.s66 27415096

[15] Craig CL , Marshall AL, Sjöström M, Bauman AE, Booth ML, Ainsworth BE, et al International physical activity questionnaire: 12-country reliability and validity. Med Sci Sports Exerc 2003;35:1381–95. 12900694 10.1249/01.MSS.0000078924.61453.FB

[16] Ong KL , Ding J, McClelland RL, Cheung BMY, Criqui MH, Barter PJ, et al Relationship of pericardial fat with biomarkers of inflammation and hemostasis, and cardiovascular disease: the Multi-Ethnic Study of Atherosclerosis. Atherosclerosis 2015;239:386–92. 25682037 10.1016/j.atherosclerosis.2015.01.033 PMC4361311

[17] Ong K-L , Ding J, McClelland RL, Cheung BMY, Criqui MH, Barter PJ, et al Relationship of pericardial fat with lipoprotein distribution: the Multi-Ethnic study of atherosclerosis. Atherosclerosis 2015;241:664–70. 26117404 10.1016/j.atherosclerosis.2015.06.027 PMC4510019

[18] Aung N , Sanghvi MM, Piechnik SK, Neubauer S, Munroe PB, Petersen SE. The effect of blood lipids on the left ventricle. J Am Coll Cardiol 2020;76:2477–88. 33213727 10.1016/j.jacc.2020.09.583 PMC7613249

[19] Yu Q . Li B. mma: an R package for mediation analysis with multiple mediators. J Open Res Softw 2017;5:11.

[20] Van Hout MJP , Dekkers IA, Westenberg JJM, Schalij MJ, Scholte AJHA, Lamb HJ. The impact of visceral and general obesity on vascular and left ventricular function and geometry: a cross-sectional magnetic resonance imaging study of the UK Biobank. Eur Heart J Cardiovasc Imaging 2020;21:273–81. 31722392 10.1093/ehjci/jez279 PMC7031704

[21] Mahmoud I , Dykun I, Kärner L, Hendricks S, Totzeck M, Al-Rashid F, et al Epicardial adipose tissue differentiates in patients with and without coronary microvascular dysfunction. Int J Obes 2021;45:2058–63. 10.1038/s41366-021-00875-6 PMC8380538 34172829

[22] Nelson AJ , Worthley MI, Psaltis PJ, Carbone A, Dundon BK, Dundon RF, et al Validation of cardiovascular magnetic resonance assessment of pericardial adipose tissue volume. J Cardiovasc Magn Reson 2009;11:1–8. 19416534 10.1186/1532-429X-11-15 PMC2684106

[23] Mahajan R , Kuklik P, Grover S, Brooks AG, Wong CX, Sanders P, et al Cardiovascular magnetic resonance of total and atrial pericardial adipose tissue: a validation study and development of a 3 dimensional pericardial adipose tissue model. J Cardiovasc Magn Reson 2013;15:73. 24498950 10.1186/1532-429X-15-73 PMC3765997

[24] Rosito GA , Massaro JM, Hoffmann U, Ruberg FL, Mahabadi AA, Vasan RS, et al Pericardial fat, visceral abdominal fat, cardiovascular disease risk factors, and vascular calcification in a community-based sample the framingham heart study. Circulation 2008;117:605–13. 18212276 10.1161/CIRCULATIONAHA.107.743062

[25] Al-Talabany S , Mordi I, Graeme Houston J, Colhoun HM, Weir-McCall JR, Matthew SZ, et al Epicardial adipose tissue is related to arterial stiffness and inflammation in patients with cardiovascular disease and type 2 diabetes. BMC Cardiovasc Disord 2018;18:1–8. 29433433 10.1186/s12872-018-0770-z PMC5809843

[26] Kim JS , Kim SW, Lee JS, Lee SK, Abbott R, Lee KY, et al Association of pericardial adipose tissue with left ventricular structure and function: a region-specific effect? Cardiovasc Diabetol 2021;20:26. 33494780 10.1186/s12933-021-01219-4 PMC7836147

[27] Mahabadi AA , Lehmann N, Möhlenkamp S, Pundt N, Dykun I, Roggenbuck U, et al Noncoronary measures enhance the predictive value of cardiac CT above traditional risk factors and CAC score in the general population. JACC Cardiovasc Imaging 2016;9:1177–85. 27450878 10.1016/j.jcmg.2015.12.024

[28] Mahabadi AA , Anapliotis V, Dykun I, Hendricks S, Al-Rashid F, Lüdike P, et al Epicardial fat and incident heart failure with preserved ejection fraction in patients with coronary artery disease. Int J Cardiol 2022;357:140–5. 35395282 10.1016/j.ijcard.2022.04.009

[29] Mazurek T , Zhang LF, Zalewski A, Mannion JD, Diehl JT, Arafat H, et al Human epicardial adipose tissue is a source of inflammatory mediators. Circulation 2003;108:2460–66. 14581396 10.1161/01.CIR.0000099542.57313.C5

[30] Iacobellis G , Bianco AC. Epicardial adipose tissue: emerging physiological, pathophysiological and clinical features. Trends Endocrinol Metab 2011;22:450–7. 21852149 10.1016/j.tem.2011.07.003 PMC4978122

[31] Xie Z , Zhu J, Li W, Liu L, Zhuo K, Yang R, et al Relationship of epicardial fat volume with coronary plaque characteristics, coronary artery calcification score, coronary stenosis, and CT-FFR for lesion-specific ischemia in patients with known or suspected coronary artery disease. Int J Cardiol 2021;332:8–14. 33775790 10.1016/j.ijcard.2021.03.052

[32] Chang KF , Lin G, Huang PC, Juan YH, Wang CH, Tsai SY, et al Left ventricular function and myocardial triglyceride content on 3T cardiac MR predict major cardiovascular adverse events and readmission in patients hospitalized with acute heart failure. J Clin Med 2020;9:1–15. 10.3390/jcm9010169 PMC7019990 31936313

[33] Raisi-Estabragh Z , McCracken C, Condurache D, Aung N, Vargas JD, Naderi H, et al Left atrial structure and function are associated with cardiovascular outcomes independent of left ventricular measures: a UK Biobank CMR study. Eur Hear J - Cardiovasc Imaging 2021:jeab266. 10.1093/ehjci/jeab266 PMC9365306 34907415

[34] Raisi-Estabragh Z , McCracken C, Gkontra P, Jaggi A, Ardissino M, Cooper J, et al Associations of meat and fish consumption with conventional and radiomics cardiovascular magnetic resonance phenotypes in the UK biobank. Front Cardiovasc Med 2021;8:667849. 34026874 10.3389/fcvm.2021.667849 PMC8133433

[35] Teijeira-Fernandez E , Eiras S, Grigorian-Shamagian L, Fernandez A, Adrio B, Gonzalez-Juanatey JR. Epicardial adipose tissue expression of adiponectin is lower in patients with hypertension. J Hum Hypertens 2008;22:856–63. 18650840 10.1038/jhh.2008.75

