# Supplementary material for: Pericardial adiposity is independently linked to adverse cardiovascular phenotypes: a CMR study of 42 598 UK Biobank participants
Source: Eur Heart J Cardiovasc Imaging. 2022 May 31;23(11):1471–81. doi: 10.1093/ehjci/jeac101 (PMC9584621; doi:10.1093/ehjci/jeac101)
Supplement: jeac101_Supplementary_Data [file jeac101_supplementary_data.docx]

**Supplementary Figure 1. Sample selection**


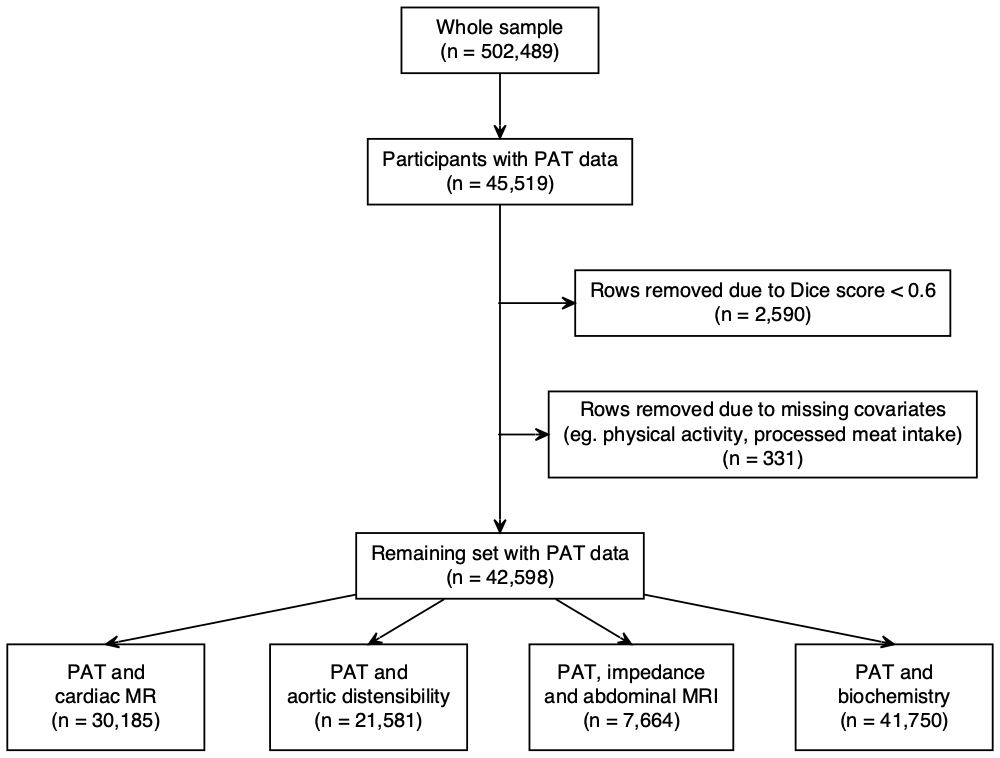


**Supplementary Figure 1 footnote.** PAT = pericardial adipose tissue, MRI = magnetic resonance imaging

**Supplementary Figure 2. Percentage of total effect mediated by each variable in the relationships between PAT and cardiovascular phenotypes from multiple mediation analysis**


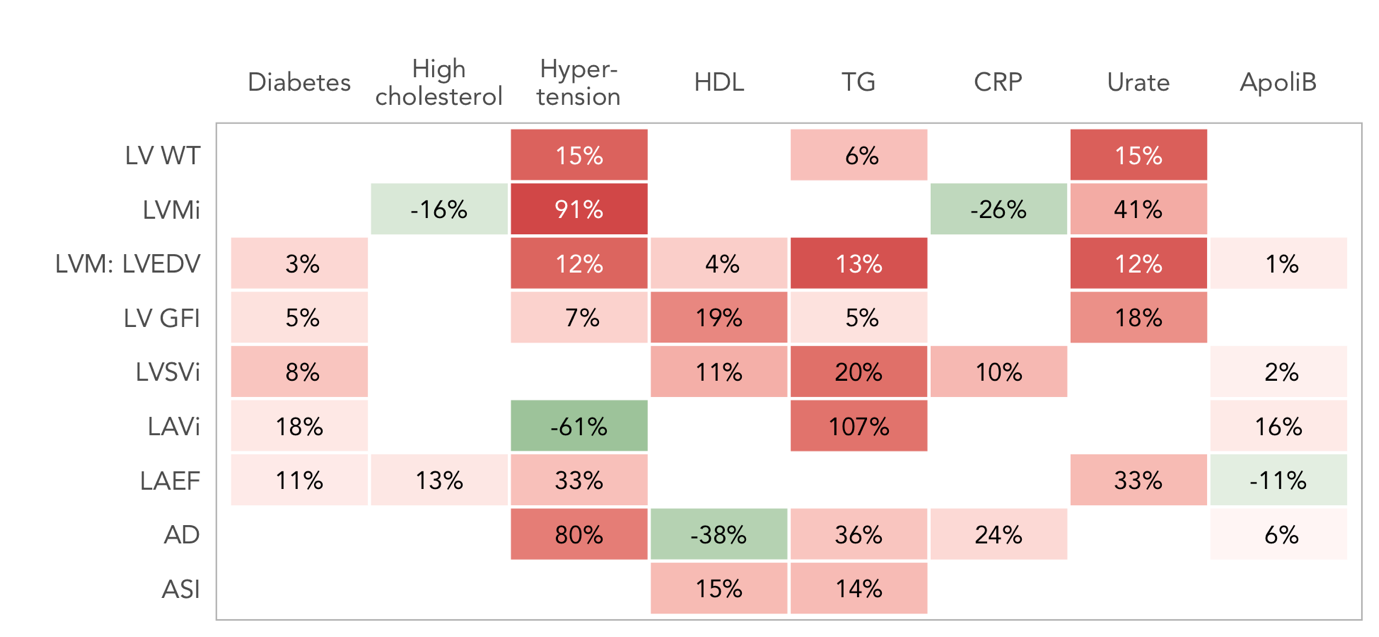


**Supplementary Figure 2 footnote.** Proportions mediated (ratio of effect to total effect) between PAT (the exposure) and cardiovascular metric for each of the 8 mediator variables modelled together. Multiple mediation models were adjusted by age, sex, ethnicity, Townsend deprivation score, physical activity, processed meat intake, smoking, body mass index, and waist-hip ratio. Analyses were conducted using the *mmabig* package in R, with n=400 bootstrapped samples. Only effects significantly different from zero are shown, ascertained where bootstrapped confidence intervals did not contain zero, and parametric p-value < 0.03. Darker colours reflect larger average effects. PAT= pericardial adipose tissue, ApoliB = Apolipoprotein B, AD= aortic distensibility, ASI = arterial stiffness index, CRP = C-reactive protein, HDL = high-density lipoprotein cholesterol, LAEF= left atrial ejection fraction, LAVi= left atrial volume index, LV left ventricular, LV GFI= LV global function index, LVMi = LV mass index, LVSVi = LV stroke volume index, LV WT = LV wall thickness, TG = serum triglycerides.

**Supplementary Table 1. Identification of diabetes, high cholesterol and hypertension**

| Source | Field ID / Code | Description |
| --- | --- | --- |
| **Diabetes** |  |  |
| Diagnosed by doctor | 2443 | Diabetes diagnosed by doctor |
|  | [2976](https://biobank.ndph.ox.ac.uk/showcase/field.cgi?id=2976) | Age diabetes diagnosed by doctor |
| Self-report (Field ID 20002) |  | Diabetes |
|  |  | Type 1 diabetes |
|  |  | Type 2 diabetes |
| ICD10 | E10 | Type 1 diabetes mellitus |
|  | E11 | Type 2 diabetes mellitus |
|  | E13 | Other specified diabetes mellitus |
|  | E14 | Unspecified diabetes mellitus |
|  | G590 | Diabetic mononeuropathy |
|  | G632 | Diabetic polyneuropathy |
|  | H280 | Diabetic cataract |
|  | H360 | Diabetic retinopathy |
|  | M142 | Diabetic arthropathy |
|  | N083 | Glomerular disorders in diabetes mellitus |
|  | O240 | Pre-existing type 1 diabetes mellitus in pregnancy |
|  | O241 | Pre-existing type 2 diabetes mellitus in pregnancy |
|  | O243 | Pre-existing diabetes mellitus, unspecified in pregnancy |
|  | O244 | Diabetes mellitus arising in pregnancy |
|  | O249 | Diabetes mellitus in pregnancy, unspecified |
|  | Y423 | Insulin and oral hypoglycaemic [antidiabetic] drugs |
| First occurrences | 130706 | Date E10 first reported |
|  | 130708 | Date E11 first reported |
|  | 130712 | Date E13 first reported |
|  | 130714 | Date E14 first reported |
| Medications (6177, 6153) | 3 | Insulin |
| Biochemistry | 30750 | Glycated haemoglobin (HbA1c) > 48 mmol/L |
| ICD9 | 250 | Diabetes mellitus |
| **High cholesterol** |  |  |
| Self-report (Field ID 20002) |  | High cholesterol |
| ICD10 | E780 | Pure hypercholesterolaemia |
|  | E782 | Mixed hyperlipidaemia |
|  | E783 | Hyperchylomicronaemia |
|  | E784 | Other hyperlipidaemia |
|  | E785 | Hyperlipidaemia, unspecified |
| First occurrences | 130814 | Date E78 first reported |
| Medications (6177, 6153) | 1 | Cholesterol lowering medication |
| Biochemistry | 30690 | Total cholesterol > 7 mmol/L |
| **Hypertension** |  |  |
| Self-report (Field ID 20002) |  | Essential hypertension |
|  |  | Hypertension |
| ICD10 | I10 | Essential (primary) hypertension |
| Medications (6177, 6153) | 2 | Blood pressure medication |
| First occurrences | 131286 | Date I10 first reported (essential (primary) hypertension) |
| Diagnosed by doctor | 6150: 4 | High blood pressure |
|  | 2966 | Age high blood pressure diagnosed |

**Supplementary Table 1 footnote.** Where source = ICD10, these were drawn from UK Biobank fields 41270, 41280, 41259, 41234, where source = ICD9, these were drawn from UK Biobank fields 41271, 41281, 41259, 41234. Where a 3-digit ICD9/ICD10 code is given, this includes all 4-digit sub-codes (for example, E10 includes E100, E101 etc.). ICD: international classification of diseases

**Supplementary Table 2. Combining body mass index, waist-hip ratio, body impedance measures, and adiposity by MRI**

| **A: Pairwise Pearson correlations between fat measures** | | | | | |
| --- | --- | --- | --- | --- | --- |
| Variable | Pericardial fat (log) | BMI (log) | Waist circ. (cm) | Waist-to-hip-ratio | Min sample size |
|  |  |  |  |  |  |
| Pericardial fat (log) | 1.000 | 0.465 | 0.589 | 0.538 | 42,598 |
|  |  |  |  |  |  |
| **Non-imaging adiposity measures** |  |  |  |  |  |
| BMI (log) | 0.465 | 1.000 | 0.807 | 0.438 |  |
| Waist circumference (cm) | 0.589 | 0.807 | 1.000 | 0.802 |  |
| Waist-hip ratio | 0.538 | 0.438 | 0.802 | 1.000 |  |
| **Impedance measures** |  |  |  |  |  |
| Whole body fat mass by impedance | 0.266 | 0.749 | 0.548 | 0.139 | 42,056 |
| Trunk fat mass by impedance | 0.399 | 0.761 | 0.673 | 0.328 |  |
| **MRI measures** |  |  |  |  |  |
| Abdominal subcutaneous (MRI) | 0.226 | 0.780 | 0.538 | 0.081 | 9,099 |
| Visceral adipose tissue (MRI) | 0.665 | 0.685 | 0.827 | 0.725 |  |
| Total adipose tissue volume (MRI) | 0.384 | 0.864 | 0.676 | 0.221 | 7,780 |

**B: Principal component loadings**

|  | Principal components (n= 7,664) | | |
| --- | --- | --- | --- |
| **Variable** | **Total** | **Visceral** | **PAT** |
| BMI (log) | **0.784** | 0.473 | 0.162 |
| Whole body fat mass by impedance | **0.947** | 0.070 | 0.104 |
| Trunk fat mass by impedance | **0.857** | 0.259 | 0.197 |
| Abdominal subcutaneous (MRI) | **0.942** | 0.066 | 0.020 |
| Total adipose tissue (MRI) | **0.921** | 0.240 | 0.162 |
| Waist circumference (cm) | 0.484 | **0.820** | 0.210 |
| Waist-hip ratio | -0.019 | **0.955** | 0.166 |
| Visceral adipose tissue (MRI) | 0.336 | **0.754** | 0.408 |
| Pericardial fat (log) | 0.159 | 0.381 | **0.901** |

**C: Principal components analysis fit indices**

| **Fit index** | **Total  (PC1)** | **Visceral  (PC2)** | **PAT  (PC3)** |
| --- | --- | --- | --- |
| Proportion variance explained | 48% | 30% | 13% |
| Cumulative proportion variance explained | 48% | 78% | 91% |
| Root mean square of residuals (RMSR) |  |  | 0.039 |
| Fit based on off-diagonal values |  |  | 0.996 |
| Test that 3 components are sufficient | Chi-square = 848.71, df = 12  p-value = 5.88 x 10 ^-174^ | | |

**Supplementary Table 2 footnote.** BMI= body mass index, MRI= magnetic resonance imaging, PAT: pericardial adipose tissue, PC= principal component. The MRI-based measures are described in the following publication: West J, et al. Feasibility of MR-Based Body Composition Analysis in Large Scale Population Studies. Gonzalez-Bulnes A, ed. *PLoS One* 2016;**11**:e0163332. The impedance obesity measures are as per UK Biobank protocol: <https://biobank.ndph.ox.ac.uk/showcase/ukb/docs/body_composition.pdf>.

**Supplementary Table 3. Associations between PAT and blood biomarkers**

| **Biomarker** | **Model 1:** | **Model 2:** | **Model 3** | **Model 4** |
| --- | --- | --- | --- | --- |
| Adjustment description | Covariate adjusted | Covariates +  BMI and WHR | Covariates, BMI, WHR, and VRFs | Covariates, VRFs and PC scores |
| Sample size [min – max] | [32,015– 40,548] | | | [5,771– 7,282] |
| Total cholesterol | 0.02* | 0.02* | 0.02* | 0.03* |
|  | [0.01, 0.04] | [0.01, 0.03] | [0.00, 0.03] | [0.00, 0.05] |
| HDL cholesterol | -0.18* | -0.04* | -0.04* | -0.11* |
|  | [-0.19, -0.17] | [-0.05, -0.03] | [-0.05, -0.03] | [-0.13, -0.08] |
| LDL cholesterol | 0.06* | 0.02* | 0.02* | 0.03* |
|  | [0.05, 0.07] | [0.01, 0.03] | [0.01, 0.03] | [0.01, 0.06] |
| Triglycerides | 0.22* | 0.07* | 0.07* | 0.16* |
|  | [0.21, 0.23] | [0.06, 0.08] | [0.06, 0.08] | [0.14, 0.19] |
| Lipoprotein A | 0.00 | 0.00 | -0.00 | -0.02 |
|  | [-0.01, 0.02] | [-0.01, 0.01] | [-0.01, 0.01] | [-0.05, 0.01] |
| Apolipoprotein A | -0.13* | -0.02* | -0.02* | -0.07* |
|  | [-0.14, -0.12] | [-0.03, -0.01] | [-0.03, -0.01] | [-0.09, -0.04] |
| Apolipoprotein B | 0.09* | 0.03* | 0.03* | 0.06* |
|  | [0.08, 0.10] | [0.02, 0.04] | [0.02, 0.04] | [0.03, 0.08] |
| HbA1c | 0.09* | 0.01 | 0.00 | 0.04* |
|  | [0.08, 0.10] | [-0.00, 0.02] | [-0.01, 0.01] | [0.02, 0.06] |
| Glucose | 0.07* | 0.01 | 0.01 | 0.03 |
|  | [0.06, 0.08] | [-0.00, 0.02] | [-0.01, 0.02] | [0.00, 0.05] |
| C reactive protein | 0.24* | 0.05* | 0.05* | 0.13* |
|  | [0.23, 0.25] | [0.04, 0.06] | [0.04, 0.06] | [0.11, 0.15] |
| Lymphocyte count | 0.07* | 0.02* | 0.02* | 0.04* |
|  | [0.06, 0.08] | [0.01, 0.03] | [0.00, 0.03] | [0.02, 0.07] |
| Monocyte count | 0.07* | 0.03* | 0.02* | 0.05* |
|  | [0.06, 0.08] | [0.01, 0.04] | [0.01, 0.04] | [0.02, 0.07] |
| Cystatin C | 0.15* | 0.04* | 0.04* | 0.08* |
|  | [0.14, 0.16] | [0.03, 0.05] | [0.03, 0.05] | [0.06, 0.10] |
| Urate | 0.16* | 0.02* | 0.02* | 0.08* |
|  | [0.15, 0.17] | [0.01, 0.03] | [0.01, 0.03] | [0.06, 0.09] |

**Supplementary Table 3 footnote.** Results are the association of the PAT variable (model exposure) with each blood biomarker (set as model outcome) from linear regression models expressed as standardised beta coefficients per SD increase in log PAT area (cm^2^), corresponding 95% confidence intervals (CIs), and p-values. *indicates a p-value significant with a false discovery rate of 0.05 across exposures. ^†^ indicates a p-value below the reporting threshold. Model 1 covariates: age, sex, ethnicity, Townsend score, smoking, physical activity, and processed food intake. Model 2 covariates: Model 1 + body mass index and waist-to-hip ratio. Model 3 covariates: Model 2 + diabetes, hypertension, and high cholesterol. Model 4 covariates: Model 1 + diabetes, hypertension, high cholesterol and the three obesity PCs (total, visceral, pericardial) derived from all available obesity measures (see Supplementary Table 2B).

**Supplementary Table 4: Details of multiple mediation between PAT and metrics of cardiovascular structure and function, mediated by vascular risk factors and blood biomarkers**

| **Outcome** | **Effect** | **Coef** | **Lower** | **Upper** | **Prop. mediated** | **PValue** | **Sig** |
| --- | --- | --- | --- | --- | --- | --- | --- |
| LV WT | PAT total effect | 0.1664 | 0.1453 | 0.1763 | 100.0% | 1.00x10^-66^ | * |
|  | PAT direct effect | 0.1024 | 0.0907 | 0.1142 | 61.6% | 1.16x10^-49^ | * |
|  | PAT via Diabetes | 0.0021 | 0.0003 | 0.0043 | 1.3% | 0.0366 |  |
|  | PAT via High cholesterol | -0.0022 | -0.0050 | -0.0005 | -1.3% | 0.0614 |  |
|  | PAT via Hypertension | 0.0249 | 0.0222 | 0.0278 | 15.0% | 3.87x10^-51^ | * |
|  | PAT via HDL | 0.0055 | -0.0011 | 0.0084 | 3.3% | 0.0258 |  |
|  | PAT via TG | 0.0102 | 0.0069 | 0.0142 | 6.1% | 6.28x10^-8^ | * |
|  | PAT via CRP | -0.0023 | -0.0048 | -0.0004 | -1.4% | 0.0461 |  |
|  | PAT via Urate | 0.0254 | 0.0143 | 0.0291 | 15.3% | 6.65x10^-11^ | * |
|  | PAT via Apoli B | 0.0003 | -0.0005 | 0.0013 | 0.2% | 0.4949 |  |
| LVMi | PAT total effect | 0.0329 | 0.0200 | 0.0460 | 100.0% | 1.07x10^-6^ | * |
|  | PAT direct effect | 0.0055 | -0.0053 | 0.0144 | 15.3% | 0.2779 |  |
|  | PAT via High cholesterol | -0.0049 | -0.0069 | -0.0030 | -15.6% | 1.66x10^-6^ | * |
|  | PAT via Hypertension | 0.0287 | 0.0257 | 0.0315 | 91.1% | 4.34x10^-59^ | * |
|  | PAT via HDL | -0.0005 | -0.0028 | 0.0028 | -2.1% | 0.713 |  |
|  | PAT via TG | -0.0011 | -0.0031 | 0.0020 | -3.6% | 0.4145 |  |
|  | PAT via CRP | -0.0082 | -0.0106 | -0.0059 | -26.2% | 3.57x10^-11^ | * |
|  | PAT via Urate | 0.0134 | 0.0064 | 0.0208 | 41.2% | 2.72x10^-4^ | * |
|  | PAT via Apoli B | -0.0001 | -0.0008 | 0.0008 | -0.2% | 0.8642 |  |
| LVM: | PAT total effect | 0.2109 | 0.1936 | 0.2336 | 100.0% | 5.28x10^-65^ | * |
| LVEDV | PAT direct effect | 0.1149 | 0.1018 | 0.1302 | 54.5% | 2.64x10^-44^ | * |
|  | PAT via Diabetes | 0.0064 | 0.0038 | 0.0089 | 3.0% | 1.42x10^-6^ | * |
|  | PAT via High cholesterol | 0.0007 | -0.0016 | 0.0025 | 0.3% | 0.5226 |  |
|  | PAT via Hypertension | 0.0245 | 0.0217 | 0.0278 | 11.6% | 9.30x10^-44^ | * |
|  | PAT via HDL | 0.0078 | 0.0031 | 0.0139 | 3.7% | 0.0051 | * |
|  | PAT via TG | 0.0272 | 0.0226 | 0.0316 | 12.9% | 8.41x10^-28^ | * |
|  | PAT via CRP | 0.0004 | -0.0015 | 0.0015 | 0.2% | 0.5738 |  |
|  | PAT via Urate | 0.0261 | 0.0180 | 0.0369 | 12.3% | 1.18x10^-7^ | * |
|  | PAT via Apoli B | 0.0030 | 0.0015 | 0.0043 | 1.4% | 4.38x10^-5^ | * |
| LV GFI | PAT total effect | -0.1016 | -0.1166 | -0.0875 | 100.0% | 2.91x10^-35^ | * |
|  | PAT direct effect | -0.0474 | -0.0656 | -0.0343 | 46.5% | 6.44x10^-9^ | * |
|  | PAT via Diabetes | -0.0047 | -0.0072 | -0.0025 | 4.7% | 8.97x10^-5^ | * |
|  | PAT via High cholesterol | -0.0006 | -0.0017 | 0.0015 | 0.6% | 0.497 |  |
|  | PAT via Hypertension | -0.0072 | -0.0099 | -0.0046 | 7.2% | 1.32x10^-7^ | * |
|  | PAT via HDL | -0.0192 | -0.0240 | -0.0126 | 18.9% | 1.43x10^-10^ | * |
|  | PAT via TG | -0.0047 | -0.0092 | -0.0008 | 4.6% | 0.0296 | * |
|  | PAT via Urate | -0.0178 | -0.0256 | -0.0076 | 17.6% | 1.19x10^-4^ | * |
| LVSVi | PAT total effect | -0.1156 | -0.1327 | -0.0977 | 100.0% | 2.62x10^-32^ | * |
|  | PAT direct effect | -0.0552 | -0.0716 | -0.0381 | 47.5% | 3.09x10^-10^ | * |
|  | PAT via Diabetes | -0.0094 | -0.0120 | -0.0069 | 8.1% | 2.47x10^-12^ | * |
|  | PAT via High cholesterol | -0.0016 | -0.0038 | 0.0009 | 1.4% | 0.1893 |  |
|  | PAT via HDL | -0.0128 | -0.0183 | -0.0077 | 11.1% | 2.71x10^-6^ | * |
|  | PAT via TG | -0.0228 | -0.0276 | -0.0180 | 19.9% | 6.28x10^-19^ | * |
|  | PAT via CRP | -0.0114 | -0.0142 | -0.0082 | 9.9% | 1.13x10^-12^ | * |
|  | PAT via Urate | -0.0001 | -0.0027 | 0.0027 | 0.1% | 0.9484 |  |
|  | PAT via Apoli B | -0.0024 | -0.0039 | -0.0010 | 2.1% | 0.0013 | * |
| LAVi | PAT total effect | -0.0215 | -0.0279 | -0.0123 | 100.0% | 1.35x10^-7^ | * |
|  | PAT direct effect | -0.0002 | -0.0037 | 0.0037 | 0.7% | 0.8958 |  |
|  | PAT via Diabetes | -0.0037 | -0.0064 | -0.0008 | 17.5% | 0.0092 | * |
|  | PAT via High cholesterol | -0.0003 | -0.0018 | 0.0018 | 1.6% | 0.7104 |  |
|  | PAT via Hypertension | 0.0127 | 0.0081 | 0.0152 | -61.4% | 6.57x10^-12^ | * |
|  | PAT via HDL | -0.0005 | -0.0023 | 0.0023 | 2.3% | 0.6583 |  |
|  | PAT via TG | -0.0224 | -0.0277 | -0.0170 | 106.6% | 3.43x10^-15^ | * |
|  | PAT via CRP | -0.0035 | -0.0058 | 0.0011 | 16.3% | 0.0465 |  |
|  | PAT via Apoli B | -0.0034 | -0.0052 | -0.0018 | 16.4% | 1.27x10^-4^ | * |
| LAEF | PAT total effect | -0.0330 | -0.0497 | -0.0113 | 100.0% | 8.33x10^-4^ | * |
|  | PAT direct effect | -0.0040 | -0.0169 | 0.0169 | 8.4% | 0.6407 |  |
|  | PAT via Diabetes | -0.0033 | -0.0064 | -0.0005 | 10.5% | 0.0283 | * |
|  | PAT via High cholesterol | -0.0039 | -0.0072 | -0.0009 | 12.7% | 0.0166 | * |
|  | PAT via Hypertension | -0.0101 | -0.0140 | -0.0067 | 33.2% | 8.69x10^-8^ | * |
|  | PAT via HDL | -0.0036 | -0.0089 | 0.0012 | 10.8% | 0.158 |  |
|  | PAT via CRP | -0.0008 | -0.0024 | 0.0014 | 2.3% | 0.4292 |  |
|  | PAT via Urate | -0.0109 | -0.0192 | -0.0049 | 33.5% | 0.003 | * |
|  | PAT via Apoli B | 0.0036 | 0.0019 | 0.0056 | -11.4% | 2.05x10^-4^ | * |
| AD | PAT total effect | -0.0261 | -0.0326 | -0.0209 | 100.0% | 6.12x10^-17^ | * |
|  | PAT direct effect | 0.0000 | -0.0001 | 0.0001 | 0.0% | 0.9456 |  |
|  | PAT via Diabetes | -0.0012 | -0.0035 | 0.0004 | 4.4% | 0.2483 |  |
|  | PAT via High cholesterol | 0.0022 | 0.0006 | 0.0074 | -8.6% | 0.2032 |  |
|  | PAT via Hypertension | -0.0207 | -0.0245 | -0.0181 | 80.2% | 5.65x10^-31^ | * |
|  | PAT via HDL | 0.0096 | 0.0050 | 0.0178 | -37.7% | 0.0036 | * |
|  | PAT via TG | -0.0093 | -0.0194 | -0.0058 | 36.2% | 0.0073 | * |
|  | PAT via CRP | -0.0061 | -0.0092 | -0.0035 | 23.8% | 3.13x10^-5^ | * |
|  | PAT via Urate | 0.0011 | -0.0020 | 0.0043 | -4.4% | 0.5073 |  |
|  | PAT via Apoli B | -0.0016 | -0.0029 | -0.0001 | 6.1% | 0.0276 | * |
| ASI | PAT total effect | 0.0762 | 0.0587 | 0.0882 | 100.0% | 1.24x10^-21^ | * |
|  | PAT direct effect | 0.0459 | 0.0215 | 0.0558 | 59.9% | 2.69x10^-7^ | * |
|  | PAT via Diabetes | 0.0006 | -0.0014 | 0.0016 | 0.7% | 0.4681 |  |
|  | PAT via High cholesterol | -0.0004 | -0.0016 | 0.0016 | -0.5% | 0.6437 |  |
|  | PAT via Hypertension | 0.0015 | -0.0005 | 0.0038 | 2.0% | 0.1637 |  |
|  | PAT via HDL | 0.0110 | 0.0069 | 0.0176 | 14.5% | 6.72x10-5 | * |
|  | PAT via TG | 0.0109 | 0.0074 | 0.0156 | 14.4% | 3.55x10-7 | * |
|  | PAT via CRP | 0.0013 | -0.0016 | 0.0028 | 1.7% | 0.2468 |  |
|  | PAT via Urate | 0.0049 | 0.0014 | 0.0155 | 6.5% | 0.1726 |  |
|  | PAT via Apoli B | 0.0005 | -0.0007 | 0.0012 | 0.6% | 0.3357 |  |

**Supplementary Table 4 footnote.** Coefficients from multiple mediation using the mediators shown together. All models are adjusted by age, sex, ethnicity, Townsend deprivation score, physical activity, processed meat intake, smoking, body mass index and waist-hip ratio. Multiple mediation modelling was conducted using the *mmabig* package in R, with n=400 bootstrapped samples. P-values are Student’s T approximations given bootstrapped estimates and standard deviations. AD= aortic distensibility; ASI= arterial stiffness index, LAEF = left atrium ejection fraction, LAVi= maximal left atrial volume index LV GFI = left ventricular global function index, LVM: LVEDV = left ventricular mass to left ventricular end-diastolic area ratio, LVSVi= left ventricular stroke volume index, LV WT= left ventricular wall thickness, PAT= pericardial adipose tissue.

**Supplementary Table 5. Cardiometabolic disease and blood biomarkers mediate between PAT and cardiovascular metrics**

| **A) Proportions mediated** | | | | | | | | | | | | | |
| --- | --- | --- | --- | --- | --- | --- | --- | --- | --- | --- | --- | --- | --- |
| Outcome | Total effect | PAT direct effect | PAT via Diabetes | PAT via High chol | PAT via Hypertension | PAT via HDL | PAT via TG | PAT via CRP | PAT via Urate | PAT via Apoli B | Fully mediated | Total effect is |  |
| LV WT | 100% | 62% |  |  | 15% |  | 6% |  | 15% |  | No | Pos |  |
| LVMi | 100% |  |  | -16% | 91% |  |  | -26% | 41% |  | Yes | Pos |  |
| LVM: LVEDV | 100% | 54% | 3% |  | 12% | 4% | 13% |  | 12% | 1% | No | Pos |  |
| LV GFI | 100% | 46% | 5% |  | 7% | 19% | 5% |  | 18% |  | No | Neg |  |
| LVSVi | 100% | 47% | 8% |  |  | 11% | 20% | 10% |  | 2% | No | Neg |  |
| LAVi | 100% |  | 18% |  | -61% |  | 107% |  |  | 16% | Yes | Neg |  |
| LAEF | 100% |  | 11% | 13% | 33% |  |  |  | 33% | -11% | Yes | Neg |  |
| AD | 100% |  |  |  | 80% | -38% | 36% | 24% |  | 6% | Yes | Neg |  |
| ASI | 100% | 60% |  |  |  | 15% | 14% |  |  |  | No | Pos |  |
|  |  |  |  |  |  |  |  |  |  |  |  |  |  |
|  | | | | | | | | | | | | | |
| Outcome | Total effect | PAT direct effect | PAT via Diabetes | PAT via High chol | PAT via Hypertension | PAT via HDL | PAT via TG | PAT via CRP | PAT via Urate | PAT via Apoli B | Fully mediated | Total effect is |  |
| LV WT | 0.166 | 0.102 |  |  | 0.025 |  | 0.010 |  | 0.025 |  | No | Pos |  |
| LVMi | 0.033 |  |  | -0.005 | 0.029 |  |  | -0.008 | 0.013 |  | Yes | Pos |  |
| LVM: LVEDV | 0.211 | 0.115 | 0.006 |  | 0.025 | 0.008 | 0.027 |  | 0.026 | 0.003 | No | Pos |  |
| LV GFI | -0.102 | -0.047 | -0.005 |  | -0.007 | -0.019 | -0.005 |  | -0.018 |  | No | Neg |  |
| LVSVi | -0.116 | -0.055 | -0.009 |  |  | -0.013 | -0.023 | -0.011 |  | -0.002 | No | Neg |  |
| LAVi | -0.021 |  | -0.004 |  | 0.013 |  | -0.022 |  |  | -0.003 | Yes | Neg |  |
| LAEF | -0.033 |  | -0.003 | -0.004 | -0.010 |  |  |  | -0.011 | 0.004 | Yes | Neg |  |
| AD | -0.026 |  |  |  | -0.021 | 0.010 | -0.009 | -0.006 |  | -0.002 | Yes | Neg |  |
| ASI | 0.076 | 0.046 |  |  |  | 0.011 | 0.011 |  |  |  | No | Pos |  |

**Supplementary Table 5 footnote:** A) Proportions mediated (ratio of effect to total effect) between PAT (the exposure) and cardiovascular metric for each of the 8 mediator variables modelled together. B) Average effects from mediation analysis (from the same models as panel A) expressed in standard deviation (SD) scale. Multiple mediation models were adjusted by age, sex, ethnicity, Townsend deprivation score, physical activity, processed meat intake, smoking, body mass index, and waist-hip ratio. Analyses were conducted using the mmabig package in R, with n=400 bootstrapped samples. Only effects significantly different from zero are shown, ascertained where bootstrapped confidence intervals did not contain zero, and parametric p-value < 0.03.  PAT= pericardial adipose tissue, ApoliB = Apolipoprotein B, AD= aortic distensibility, ASI = arterial stiffness index, CRP = C-reactive protein, HDL = high-density lipoprotein cholesterol, LAEF= left atrial ejection fraction, LAVi= left atrial volume index, LV left ventricular, LV GFI= LV global function index, LVMi = LV mass index, LVSVi = LV stroke volume index, LV WT = LV wall thickness, Neg= negative, PAT= pericardial adipose tissue, Pos = positive, TG = serum triglycerides.
